# The role of CSF IL-6 levels in diagnosis and outcome prediction of autoimmune encephalitis

**DOI:** 10.3389/fimmu.2026.1865851

**Published:** 2026-07-13

**Authors:** Mikito Shimizu, Taku Hoshi, Hiroyuki Sumikura, Kyoko Higashida, Isao Fukasaka, Yuki Shimada, Takuma Sato, Mai Ito, Takahiro Tomoda, Manabu Sakaguchi, Tatsusada Okuno

**Affiliations:** 1 Department of Neurology, Hyogo Prefectural Nishinomiya Hospital, Hyogo, Japan; 2 Department of Neurology, Osaka General Medical Center, Osaka, Japan; 3 Department of Neurology, Osaka University Graduate School of Medicine, Osaka, Japan

**Keywords:** autoimmune encephalitis, CSF, diagnosis, IL-6, outcome prediction

## Abstract

**Background:**

Autoimmune encephalitis (AE) is clinically heterogeneous, and its early diagnosis and prognostication remain challenging. We aimed to determine whether cerebrospinal fluid (CSF) interleukin-6 (IL-6), a marker of central nervous system inflammation, can improve AE diagnosis and predict treatment outcomes.

**Methods:**

This retrospective cohort study (April 2014–August 2024) included patients who fulfilled the criteria for at least possible AE according to the 2016 Graus criteria and had their CSF IL-6 measured. The patients with infectious encephalitis were excluded. We collected clinical, laboratory, brain MRI, and tumor screening data. In a subset of AE patients with repeat CSF testing approximately 1 month after initiation of immunotherapy, we obtained post-treatment laboratory data, hospitalization length, and modified Rankin Scale (mRS) score at discharge. Diagnostic performance was assessed using receiver operating characteristic (ROC) analyses, and multivariable regression was used to identify predictors of discharge mRS score and hospitalization length. In antibody-negative AE, we also examined the association between post-treatment CSF IL-6 (CSF IL-6_1M) and the RAPID score, a prognostic tool for antibody-negative AE.

**Results:**

The cohort comprised 55 AE cases (25 antibody-positive). ROC analysis of CSF parameters suggested that IL-6 showed the highest sensitivity, whereas the immunoglobulin G (IgG) index demonstrated the highest specificity. In an additional analysis that included CSF IL-6 as an additional inflammatory CSF parameter within the 2016 Graus criteria, the diagnostic sensitivity increased from 73% to 85% with a specificity of 88%. Among the 35 AE patients with post-treatment CSF measurements, multivariable regression showed that only CSF IL-6_1M significantly predicted both mRS score at discharge (p = 0.0444, 95% CI 0.00104-0.0702) and hospitalization length (p = 0.0143, 95% CI 0.425-3.13). Specifically, patients with CSF IL-6_1M of < 6 pg/mL had significantly shorter hospital stays than those with higher levels (generalized Wilcoxon test, p = 0.0361). CSF IL-6_1M was also significantly associated with the RAPID score (p = 0.0492).

**Conclusion:**

CSF IL-6 is a useful marker for early AE diagnosis and CSF IL-6_1M may serve as a practical biomarker for treatment response and prognosis, particularly for antibody-negative AE. Thus, CSF IL-6 and CSF IL-6_1M assessment may facilitate more precise, timely, and individualized therapeutic interventions.

## Introduction

Autoimmune encephalitis (AE) is an immune‐mediated disorder of the central nervous system characterized by the subacute onset of neuropsychiatric symptoms, seizures, memory disturbance, and movement abnormalities (1, 2). The 2016 Graus criteria provide a practical diagnostic framework, including antibody−negative phenotypes (2). Over the past decade, the discovery of neural‐surface autoantibodies—most prominently those targeting the N-methyl-D-aspartate receptor (NMDAR), α-amino-3-hydroxy-5-methyl-4-isoxazolepropionic acid receptor (AMPAR), γ-aminobutyric acid receptor (GABAR), and leucine-rich glioma-inactivated 1 (LGI1) —has markedly improved recognition of antibody‐positive AE patients (3–6). However, despite the high specificity of these antibodies, a substantial proportion of patients remain antibody-negative (possibly as high as 50%), highlighting the importance of accurate clinical history and various examinations (such as MRI, cerebrospinal fluid (CSF) parameters, and EEG) (7, 8). ^A^ prompt and accurate diagnosis of AE is critical, because delays in initiating immunotherapy correlate with poorer neurological outcomes and increased long-term disability (9, 10).

In addition to the initial diagnosis, predicting treatment response and long−term prognosis is challenging. The clinical courses range from monophasic syndromes with excellent recovery to relapsing disease or persistent cognitive impairment9. This challenge is particularly pronounced in antibody-negative AE, where heterogeneous mechanisms and the absence of a defining antibody complicate prognostication, the selection and duration of immunotherapy, and assessment of treatment response (2, 8, 11). The ability to stratify patients according to anticipated disease severity would greatly enhance individualized care, guide the intensity and duration of immunotherapy, and inform discussions with patients and families regarding expected outcomes.

In this study, we evaluated cerebrospinal fluid (CSF) interleukin−6 (IL−6) as a candidate biomarker for AE. CSF IL−6 is a well−established indicator of ongoing central nervous system inflammation in disorders such as neuropsychiatric systemic lupus erythematosus (NPSLE) (12, 13). Mechanistically, IL−6 is a pleiotropic cytokine that promotes B−cell differentiation and supports plasma cell survival. It can also disrupt blood–brain barrier integrity, processes plausibly relevant to AE pathogenesis and treatment response (14–16). By assessing the diagnostic sensitivity and specificity of CSF IL−6 and its prognostic value for functional recovery, we aimed to address current gaps in early detection and outcome prediction in AE and to facilitate more precise and timely therapeutic strategies.

## Methods

### Study population

We screened 128 patients evaluated at Osaka General Medical Center (OGMC) between April 2014 and August 2024. Inclusion criteria were: (1) fulfillment of the 2016 Graus criteria for at least possible AE after reasonable exclusion of infectious causes and other non-AE etiologies (e.g., NPSLE, primary CNS vasculitis, febrile infection-related epilepsy syndrome (FIRES)); (2) availability of CSF IL-6 measurement and brain MRI; and (3) testing for AE-associated autoantibodies to allow classification of antibody-negative probable autoimmune encephalitis (ANPRA). Hashimoto’s encephalopathy (HE) and Bickerstaff brainstem encephalitis (BBE) were not included as antibody-negative AE, according to a previous report (8). The final diagnosis of AE or non-AE was established by comprehensive clinical adjudication based on clinical presentation, disease course, neurological examination, MRI, EEG, conventional CSF findings, tumor screening, antibody testing, treatment response, and longitudinal follow-up. CSF IL-6 values were not used as a decisive criterion for the final clinical diagnosis. To mitigate incorporation bias, patients reclassified as ANPRA after the inclusion of CSF IL-6 as an additional inflammatory CSF parameter were re-adjudicated by an independent neuroimmunologist based on information other than CSF IL-6. From 35 patients who underwent repeat CSF testing approximately 1 month after the initiation of immunotherapy, we also collected laboratory data, hospitalization length, and modified Rankin Scale (mRS) score at discharge.

### Data collection

Data on demographics (sex and age), clinical symptoms, laboratory findings, EEG, brain MRI findings, treatments, tumor survey results and neurological severity at admission and discharge were collected from the patients’ medical records. Neurological disability was assessed with the mRS score. CSF studies included cell count, protein concentration, IgG index, oligoclonal band (OCB) and IL-6 concentration. All IL-6 levels were measured using a high-sensitivity electrochemiluminescence immunoassay (ECLIA). Based on prior NPSLE evaluations, an elevated CSF IL-6 level was defined as > 4.3 pg/mL. Baseline CSF IL-6 was measured before first-line immunotherapy in all but three patients. To evaluate the incremental diagnostic value of CSF IL-6, an elevated CSF IL-6 level (> 4.3 pg/mL) was treated as an additional “inflammatory CSF” finding within the 2016 Graus criteria, together with CSF pleocytosis, oligoclonal bands, and an elevated CSF IgG index. The diagnostic sensitivity and specificity for AE were then recalculated and compared with those obtained using the original 2016 Graus criteria. To examine the potential influence of AE heterogeneity, baseline CSF IL-6 levels were stratified by AE subtype in a supplementary analysis. Subtypes comprising only one or two patients were grouped as “Other antibody-positive encephalitis.

Cell and IL-6 levels in the CSF were also evaluated one month after the start of treatment (CSF Cell_1M and CSF IL-6_1M). For prognostic analyses, an exploratory threshold of 6 pg/mL was selected based on the median CSF IL-6_1M value (5.6 pg/mL) in the antibody-negative AE subgroup to ensure comparable group sizes for Kaplan–Meier analysis. Because hospitalization length is a time-to-event outcome rather than a binary diagnostic outcome, the CSF IL-6_1M threshold was not derived from receiver operating characteristic (ROC) or Youden index-based optimization.

### Treatment profiles

First-line immunotherapy consisted of high-dose intravenous methylprednisolone (IVMP), intravenous immunoglobulin (IVIG), and plasma exchange (PLEX). Second-line modalities included rituximab and cyclophosphamide, and third-line therapies included tocilizumab. Treatment timing, number of courses, and dosing were determined by the treating physicians according to disease severity, response to prior therapy, and safety considerations. IVMP was typically administered at a dose of 1 g/day for 3–5 consecutive days. IVIG was administered at 0.4 g/kg/day for 5 days (total 2 g/kg). PLEX was performed in 5–7 sessions. Reduced or split dosing was considered for patients at higher risk of hematologic or infectious complications.

### Statistical analysis

The diagnostic performance of CSF IL-6 for classifying AE versus non-AE conditions was evaluated using ROC curves and the area under the curve (AUC). We used the generalized Wilcoxon test to compare hospitalization length based on CSF IL-6_1M levels. For univariable analysis, Student’s t-test, Mann-Whitney U test and Fisher’s exact test were used to compare two groups of continuous and categorical data, respectively. The Kruskal-Wallis test was used to assess differences in distribution among three or more groups. To identify prognostic factors, we constructed univariable and multivariable regression models for the mRS score at discharge, and a multivariable regression model for hospitalization length. For the assessment of treatment response in the univariable regression models, patients were classified into good-response (mRS score ≤ 2) and poor-response (mRS score ≥ 3) groups To assess multicollinearity, we confirmed that the variance inflation factor (VIF) was less than 5. Pearson’s correlation was used to assess the association between CSF IL-6_1M and the RAPID score (refractory status epilepticus; age of onset ≥60 years, antibody-negative probable autoimmune encephalitis subtype, infratentorial involvement and delay of immunotherapy) (8). Statistical analyses were performed with R (v4.5.1) and GraphPad Prism (v10.4.2). Data are presented as mean (range), median (range/interquartile range) or number (percentage), unless otherwise specified. Two-sided p < 0.05 was considered statistically significant.

## Results

### Diagnostic utility of CSF IL-6 for AE

**Figure 1:**
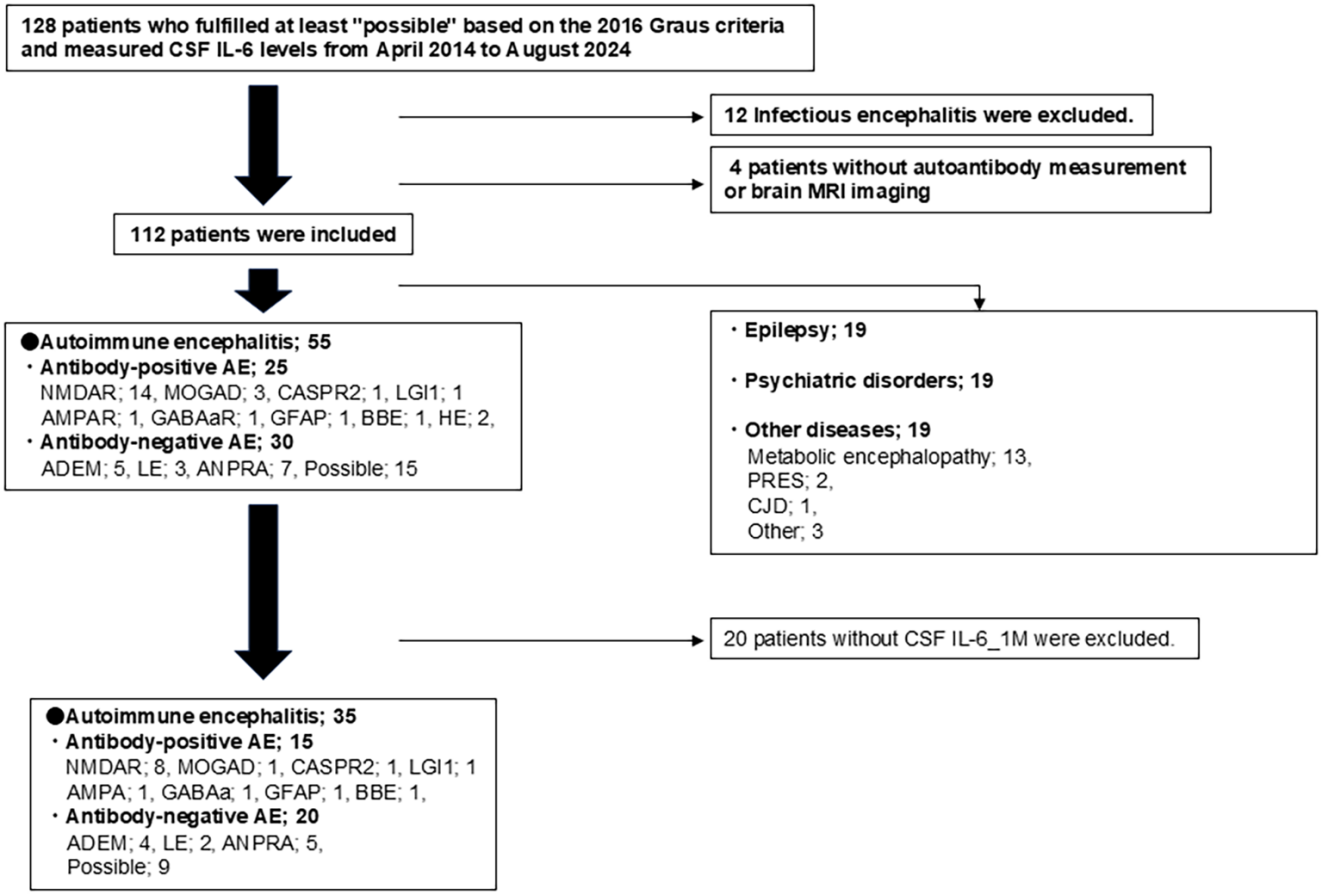
The flowchart of patient selection. Of the 112 eligible patients, 55 had autoimmune encephalitis (AE; 25 antibody-positive, 30 antibody-negative) and 57 had non-AE diagnoses (19 epilepsy, 19 primary psychiatric disorders, and 19 other conditions). For the prognostic analyses, a predefined subset of 35 AE patients who underwent CSF IL-6_1M was evaluated (15 antibody-positive, and 20 antibody-negative). AE, autoimmune encephalitis; CSF, cerebrospinal fluid; NMDAR, N-methyl-D-aspartate receptor antibody encephalitis; MOGAD, myelin-oligodendrocyte glycoprotein antibody–associated disease; CASPR2, contactin-associated protein-like 2 antibody encephalitis; LGI1, leucine-rich glioma-inactivated 1; AMPAR, α-amino-3-hydroxy-5-methyl-4-isoxazolepropionic acid receptor antibody encephalitis; GABAR, γ-aminobutyric acid receptor; GFAP, glial fibrillary acidic protein; BBE, Bickerstaff brainstem encephalitis; HE, Hashimoto’s encephalopathy; ADEM, acute disseminated encephalomyelitis; LE, limbic encephalitis; ANPRA, antibody-negative probable autoimmune encephalitis; PRES, posterior reversible encephalopathy syndrome; CJD, Creutzfeldt–Jakob disease; CSF IL-6_1M, CSF interleukin-6 measured 1 month after initiation of treatment.

Of the 128 patients who fulfilled at least “possible AE” by the 2016 Graus criteria and had CSF IL-6 measured between April 2014 and August 2024, 12 with infectious encephalitis and 4 lacking antibody testing or brain MRI were excluded (**Figure 1**). The final cohort comprised 112 patients: 55 with AE (25 antibody-positive, and 30 antibody-negative) and 57 with non-AE diagnoses (19 epilepsy, 19 primary psychiatric disorders, and 19 other conditions). These ANPRA diagnoses were reviewed by an additional neuroimmunologist and remained unchanged. Baseline comparisons of clinical characteristics, laboratory data, brain MRI findings, tumor screening results, antibodies and epileptiform discharge across AE, epilepsy, psychiatric disorders, and other diagnoses are summarized in **Table 1**. First-line treatment was initiated at a median of 2 days (IQR 0-6) after the initial CSF examination. Four patients did not receive first-line treatment. In one patient, the details of the timing of the initial treatment were unknown because the patient had been transferred from another hospital. Second-line treatment was administered in two patients and third-line treatment in only one patient, initiated 16, 52, and 128 days after diagnosis, respectively. Baseline CSF IL-6 was not significantly different between the antibody-positive and antibody-negative AE groups (median 92.8 vs 54.1 pg/mL; Mann-Whitney U test, p = 0.628; **Supplementary Table 1**). No clear correlation was also observed between AE subtype and CSF IL-6 level (Kruskal-Wallis test, p = 0.432; **Supplementary Table 1**).

**Table 1:** Baseline characteristics across diagnostic groups.

	AE (n=55)	Epilepsy (n=19)	Psychiatric disease (n=19)	Other diseases (n=19)
Age (y)	46.5 (12-88)	65.2 (16-93)	53.6 (16-81)	65.4 (23-91)
Sex (M:F)	26:29	7:12	5:14	9:10
CSF Cell	31.7 (0-170)	4.2 (0-50)	2.6 (0-18)	2.9 (0-16.7)
CSF Protein	65.8 (8-312)	46.9 (16-76)	52.8 (18-91)	48.3 (8-109)
CSF IgG index	0.67 (0.33-2.85)	0.48 (0.39-0.61)	0.50 (0.39-0.78)	0.49 (0.35-0.64)
CSF OCB	36.8%	27%	5.9%	7.1%
CSF IL-6	250 (2.2-2220)	76.2 (2.7-436)	3.8 (1.6-5.6)	61.4 (1.5-371)
MRI change	80%	31.6%	10.5%	31.6%
Tumor	17%	6.7%	7.7%	16.7%
Antibodies	45%	0%	0%	0%
ED	19%	32%	0%	6.7%

Baseline clinical characteristics, laboratory findings, brain MRI features, tumor screening results, autoantibodies and epileptiform discharges in patients with autoimmune encephalitis (AE), epilepsy, primary psychiatric disorders, and other conditions. Values are shown as mean (range) or n (%), as appropriate.

AE, autoimmune encephalitis; MRI, magnetic resonance imaging; OCB, oligoclonal band; ED, epileptiform discharge.

**Figure 2:**
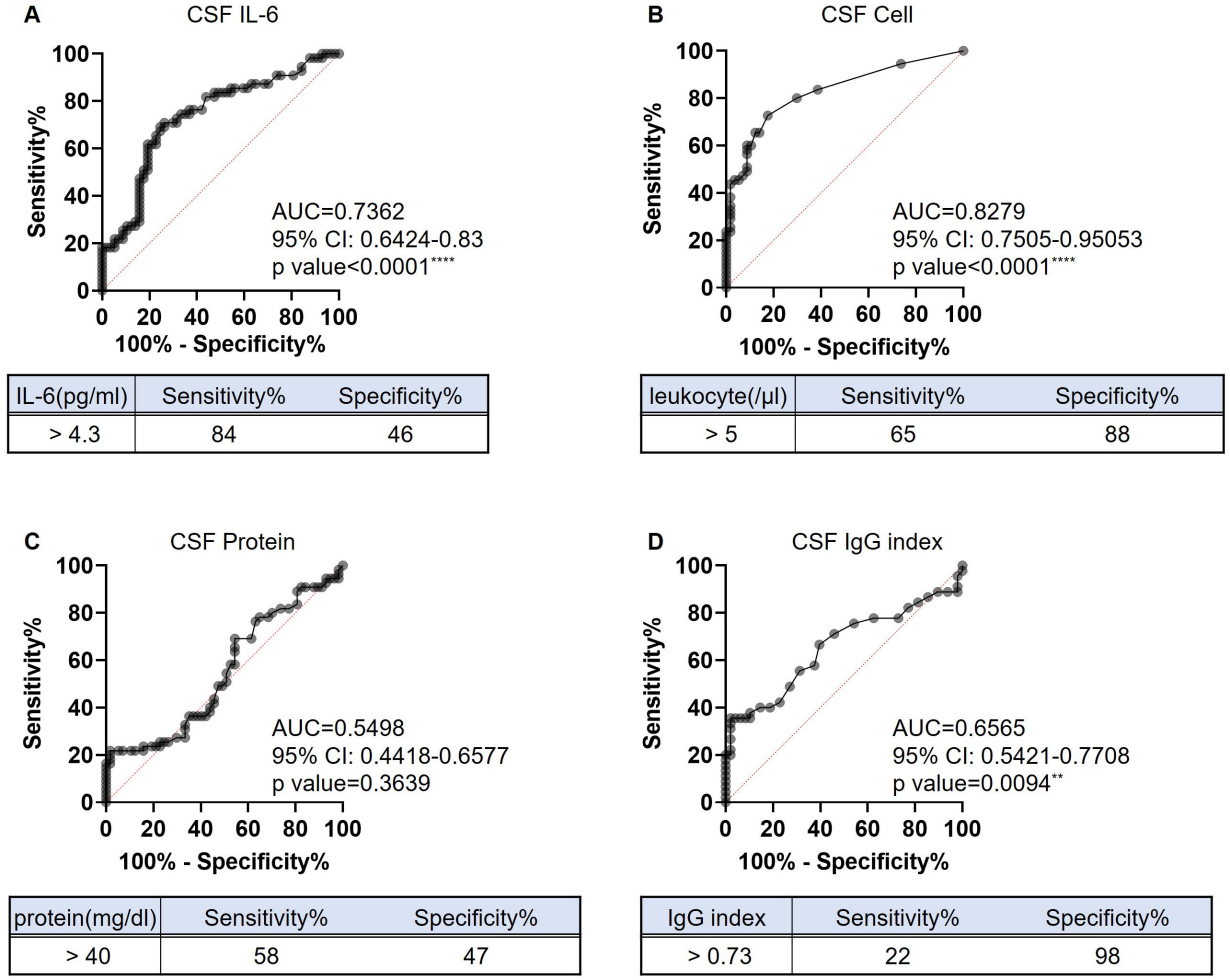
ROC curves for diagnosis of AE. Diagnostic value of **(A)** CSF IL-6, **(B)** CSF Cell, **(C)** CSF protein, and **(D)** CSF IgG index. 95% CIs were provided for the AUC values. CSF, cerebrospinal fluid; AUC, area under the curve; CI, confidence interval. (**p < 0.01; ****p < 0.0001).

ROC analyses of the CSF parameters are shown in **Figures 2A–D**. The AUC estimates and corresponding p values indicated that IL-6 (**Figure 2A**) and IgG index (**Figure 2D**) were the most informative individual markers for AE classification. Using a CSF IL-6 cutoff value of 4.3 pg/mL, the ROC analysis showed sensitivities/specificities were 84%/46% for CSF IL-6, 65%/88% for CSF cell, 58%/47% for CSF protein and 22%/98% for CSF IgG index, respectively. Using the 2016 Graus criteria, the sensitivity and specificity for distinguishing probable from possible AEs in our cohort were 73% and 96%, respectively (2). When CSF IL-6 was added to the Graus criteria, sensitivity increased to 85% with a specificity of 88%, by increasing the number of patients classified as ANPRA.

### Prognostic value of post-treatment CSF IL-6 (CSF IL-6_1M) in AE

The prognostic analysis included a subset of 35 AE patients (15 antibody-positive, 20 antibody-negative) who underwent repeat CSF testing approximately 1 month after initiation of immunotherapy. Repeat testing occurred at a median of 26.5 days (interquartile range: 14–39 days, range: 0–61 days) after the initiation of first-line immunotherapy, and prior to any second-line therapy.

On univariable analyses comparing good-response and poor-response groups, age, sex, MRI changes, and CSF IL-6_1M differed significantly. In multivariable regression analysis, sex, antibody status, and CSF IL-6_1M were independent predictors of discharge mRS score. For hospitalization length, CSF IL-6_1M was the only variable independently associated with this outcome. CSF IL-6_1M was the only variable that remained significant across the univariable and multivariable analyses for mRS score (**Table 2**) and hospitalization length (**Table 3**).

**Table 2:** Univariable and multivariable regression analyses for predictors of discharge mRS score.

	All (n=35)	mRS score				
		Univariate analysis			Multivariable analysis	
		Good (n=12)	Poor (n=23)	p-value	Coefficient (95%CI)	p-value
Age (y)	49.3 (15-88)	38.9 (18-77)	54.8 (15-88)	0.0244*	0.0280 (-0.0127-0.0687)	0.160
Male sex	51.4%	25%	65.2%	0.0354*	2.06 (1.05-3.07)	0.000813***
CSF Cell	35.6 (0-170)	61.1 (0-162)	22.3 (0-170)	0.0526	-0.00636 (-0.0237-0.0110)	0.440
CSF IgG index	0.71 (0.35-2.85)	0.71 (0.35-1.51)	0.72 (0.37-2.85)	0.974	0.493 (-1.83-2.81)	0.651
CSF OCB	44%	40%	47%	0.999	0.103 (-1.06-1.27)	0.851
CSF IL-6	252.2 (2.7-2220)	304.1 (2.7-2220)	225.1 (3.7-1570)	0.696	0.000395 (-0.00085-0.0016)	0.502
CSF Cell_1M	8.4 (0-50)	10.2 (0-37)	7.5 (0-50)	0.513	0.0187 (-0.0337-0.0710)	0.452
CSF IL-6_1M	10.5 (2.4-64.3)	4.2 (2.4-8)	13.8 (2.5-64.3)	0.02*	0.0356 (0.00104-0.0702)	0.0444*
MRI	77%	58%	87%	0.0047**	0.424 (-0.968-1.82)	0.520
Tumor	23%	42%	13%	0.0912	-1.17 (-2.78-0.447)	0.141
Antibodies	43%	50%	39%	0.721	1.60 (0.326-2.87)	0.0180*
ED	11%	8.33%	13.0%	0.999	1.07 (-0.791-2.93)	0.234

Associations between clinical, imaging, and CSF variables and mRS score at discharge are shown. CSF IL-6_1M and sex were statistically significant variables in both the univariable analyses and multivariable models for mRS score.

CSF IL-6_1M, CSF interleukin-6 measured 1 month after treatment initiation; mRS score, modified Rankin Scale; MRI, magnetic resonance imaging; ED, epileptiform discharge. (*p < 0.05; **p < 0.01; ***p < 0.001).

**Table 3:** Multivariable regression analyses for predictors of hospitalization length.

	All (n=35)	Hospitalization length	
		Multivariable analysis	
		Coefficient (95%CI)	p-value
Age	49.3 (15-88)	0.133 (-1.46-1.73)	0.858
Male sex	51.4%	34.5 (-5.10-74.0)	0.0820
CSF Cell	35.6 (0-170)	0.0747 (-0.605-0.754)	0.815
CSF IgG index	0.71 (0.35-2.85)	-2.41 (-93.1-88.3)	0.955
CSF OCB	44%	30.4 (-15.2-75.9)	0.172
CSF IL-6	252.2 (2.7-2220)	-0.00429 (-0.0530-0.0444)	0.851
CSF Cell_1M	8.4 (0-50)	-0.0192 (-2.07-2.03)	0.984
CSF IL-6_1M	10.5 (2.4-64.3)	1.78 (0.425-3.13)	0.0143*
MRI change	77%	53.7 (-0.691-108)	0.0525
Tumor	23%	3.33 (-59.7-66.4)	0.910
Antibodies	43%	40.5 (-9.20-90.2)	0.101
ED	11%	15.7 (-57.1-88.5)	0.648

Associations between clinical, imaging, and CSF variables and hospitalization length are shown. CSF IL-6_1M was the only variable that remained statistically significant in multivariable models of hospitalization length.

CSF IL-6_1M, CSF interleukin-6 measured 1 month after treatment initiation; mRS score, modified Rankin Scale; MRI, magnetic resonance imaging; ED, epileptiform discharge. (*p < 0.05).

### Antibody-negative AE subgroup analyses

**Figure 3:**
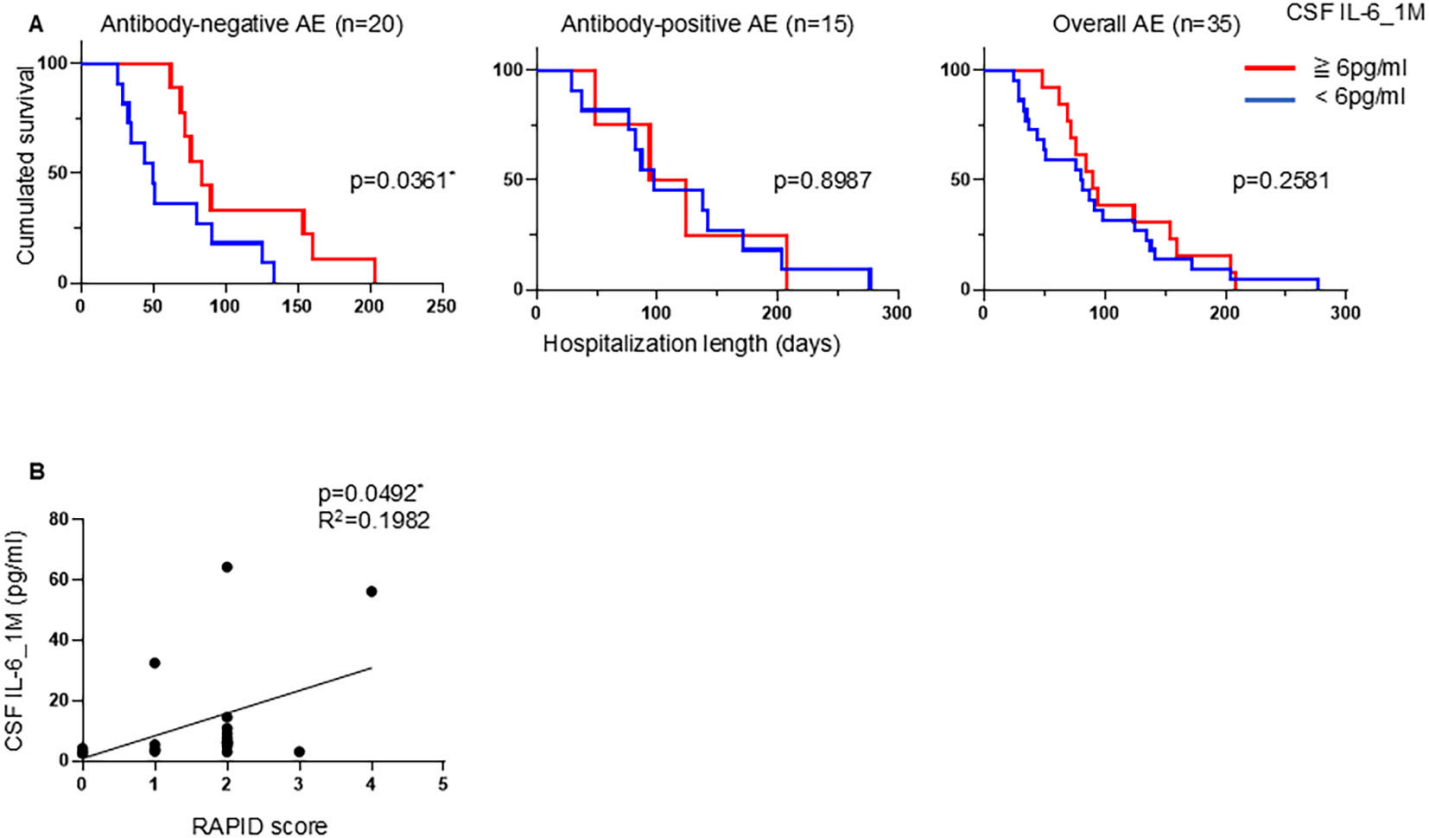
Prognostic value of post-treatment CSF IL-6 (CSF IL-6_1M) in antibody-negative autoimmune encephalitis. **(A)** Kaplan–Meier curves for time to discharge stratified by CSF IL-6_1M using a threshold of 6 pg/mL. Among antibody-negative AE, patients with lower CSF IL-6_1M (<6 pg/mL) had significantly shorter hospitalization length than those with higher levels (median 50 vs 84 days; generalized Wilcoxon test p = 0.0361). No significant differences were observed between high and low CSF IL-6_1M groups in the overall AE cohort or in antibody-positive AE. **(B)** Association between CSF IL-6_1M and the RAPID score, demonstrating a significant correlation (p = 0.0492; R² = 0.1982). CSF, cerebrospinal fluid; AE, autoimmune encephalitis; CSF IL-6_1M, CSF interleukin-6 measured 1 month after treatment initiation. (*p < 0.05).

In antibody-negative AE, patients with CSF IL-6_1M < 6 pg/mL had a significantly shorter hospitalization length than those with higher levels (median 50 vs 84 days; generalized Wilcoxon test, p = 0.0361; **Figure 3A**). No significant differences were observed in antibody-positive AE (median 98 vs 109 days; p = 0.8987) or the overall AE cohort (median 81 vs 90 days; p = 0.2581). Finally, CSF IL-6_1M was significantly correlated with the RAPID score, a prognostic tool for antibody-negative AE (8) (p = 0.0492, R² = 0.1982) (**Figure 3B**).

## Discussion

Diagnosing AE remains challenging because of its heterogeneous clinical syndromes and broad spectrum of mimicking conditions. Recent multicenter data highlighted a substantial rate of misdiagnosis in adults with presumed AE and underscored the necessity of antibody interpretation (17, 18). Best practice statements and methodological guidance on neural antibody testing emphasize the integration of serology with syndromic features, CSF/MRI/EEG findings, and longitudinal assessment (19).

Against this background, our study suggests that incorporating CSF IL−6 as an inflammatory CSF parameter significantly increases the sensitivity of the diagnostic algorithm without an unacceptable loss of specificity. This observation is biologically plausible, because IL−6 is a multifunctional cytokine that promotes B–cell differentiation, plasma cell survival, and disruption of the blood–brain barrier, all of which are pivotal in AE pathogenesis (14–16, 20, 21). Indeed, previous works have demonstrated a distinctive intrathecal activation of IL−6 and IL−17 pathways in anti−NMDAR encephalitis (22–24). Another study showed that elevated acute-phase IL-6 CSF/serum ratios in immunotherapy-naive patients with anti-LGI1 encephalitis are associated with disease severity (25). Moreover, reports demonstrating that anti-IL-6 therapy, such as tocilizumab, is effective in refractory autoimmune encephalitis further support the pathogenic role of IL-6 in the disease process (26, 27). Nevertheless, IL−6 lacks disease specificity and is elevated in patients with infectious encephalitis. In our cohort, the specificity of CSF IL-6 alone was low (46%) and may be even lower in populations in which infection has not yet been excluded. Accordingly, CSF IL-6 should be interpreted only after infectious encephalitis has been excluded by independent means (e.g., CSF PCR/culture).

Prognostication in AE has advanced with tools such as the NEOS (anti-NMDAR Encephalitis One-Year Functional Status) score (28), biomarker studies that relate intrathecal elevation of CXCL13 (29), or a B-cell–attracting chemokine, and antibody expansions (30) to outcomes. However, accurate prediction of the clinical course in AE remains elusive. In this study, CSF IL-6_1M was the only variable that consistently predicted both functional status at discharge and hospitalization length. Conceptually, CSF IL-6_1M may function as a “biological response marker,” capturing intrathecal inflammation beyond that detected by clinical or imaging measures.

Moreover, our study provides important insights into the diagnosis and prognostication of antibody-negative AE, where the unmet clinical need is greatest. Antibody-negative AE, which accounts for 40–50% of cases (8), has a heterogeneous pathology, making diagnosis more challenging. In our cohort, we found that the inclusion of CSF IL-6 > 4.3 pg/mL as one of the inflammatory CSF parameters in the 2016 Graus criteria increased the diagnostic sensitivity for AE from 73% to 85%, by increasing the number of patients classified as ANPRA. Previous studies of non-inflammatory neurological controls have generally reported very low CSF IL-6 concentrations (mean ± SD, 2.53 ± 0.43 (31) pg/mL; medians [interquartile ranges], 3.1 [2.7, 4.1] (32) pg/mL; mean ± SD, 2.2 ± 1.1 (33) pg/mL), supporting the biological plausibility that 4.3 pg/mL represents an abnormal elevation rather than a normal variation. This result suggests that in antibody-negative patients, CSF IL−6 may be a key objective marker for triggering early immunotherapy. However, it should be noted that the sensitivity and specificity reported here were achieved only when CSF IL-6 was applied within the Graus framework (i.e., in combination with other diagnostic elements). Therefore, CSF IL-6 should be used solely as an adjunct to enhance the sensitivity of the Graus framework. In addition, AE is biologically and clinically heterogeneous, and the possibility cannot be excluded that this imbalance has led to an overestimation of the diagnostic or prognostic value of CSF IL-6 levels. Although the table of AE subtypes and CSF IL-6 (**Supplementary Table 1**) did not suggest a clear subtype-specific dominance on the results (Kruskal-Wallis test, p = 0.432), an accurate assessment would require studies with larger sample sizes.

Moreover, validated prognostic frameworks also remain limited (8, 34) in antibody-negative AE. We showed that lower CSF IL-6_1M was associated with shorter hospitalization specifically in antibody-negative AE, and that CSF IL-6_1M was correlated with RAPID. These results suggested that dynamic CSF IL−6 may be informative for prognostication and refine risk stratification where antibodies associated with AE are absent. These data may help guide the intensity and duration of immunotherapy in antibody-negative cases. A possible reason why CSF IL-6 was more useful in antibody-negative AE is that IL-6 dynamics differ substantially according to antibody subtype; thus, pooling these subtypes obscures the overall trend. For example, CSF IL-6 is known to be elevated in anti-NMDAR encephalitis (32) but not markedly so in anti-LGI1 encephalitis (22, 25). To clarify subtype-specific IL-6 dynamics, future studies using large-scale cohorts are required. Alternatively, the antibody-negative group may include patients with unidentified antibodies whose IL-6 levels tend to be elevated, as in anti-NMDAR encephalitis. The latter hypothesis may be more consistent with previous reports that IL-6 promotes B-cell differentiation and enhances plasma-cell survival (14, 15, 20, 21). Therefore, the discovery of novel antibodies is necessary to clarify this point.

This study has several limitations. This was a single−center retrospective cohort study with a modest sample size. This study population was clinically and biologically heterogeneous and included patients with various forms of antibody-positive and antibody-negative AE. The pooling of these conditions may obscure subtype-specific CSF IL-6 dynamics. Individual AE subtypes should be evaluated separately in larger cohorts. Second, the IL−6 cutoff chosen in this study requires prospective validation in larger, multicenter cohorts. Third, future studies should evaluate long-term clinical outcomes at extended follow-up periods (e.g., 6 and 12 months) to clarify the relationship between IL-6 dynamics and sustained recovery or relapse risk. Fourth, the discharge mRS score is a short-term outcome measure and may not fully capture the prolonged recovery trajectory of AE. Fifth, the retrospective design cannot fully exclude incorporation bias, and the observed increase in sensitivity should be interpreted as an exploratory analysis, rather than a definitive validation of a modified diagnostic criterion. Therefore, prospective validation in an independent cohort of consecutive patients with suspected encephalitis before diagnostic classification is required. Finally, future studies should evaluate serial measurements and explore whether other CSF cytokines (e.g., IL−17, CXCL13), in combination with IL−6, further refine diagnostic and prognostic accuracy.

In summary, our findings suggest that CSF IL−6 is a valuable biomarker for both early diagnosis and prognostic stratification of AE, with particular benefit in antibody-negative cases where objective guides to therapy are limited. The incorporation of CSF IL−6 assessment into routine AE work−ups may facilitate more precise, timely, and individualized therapeutic interventions.

## Data Availability

The original contributions presented in the study are included in the article/**Supplementary Material**. Further inquiries can be directed to the corresponding author.

## Supplementary material

Supplementary Table 1 Distribution of CSF IL-6 levels across encephalitis subtypes. Associations between the subtypes of AE and CSF IL-6 are shown. CSF IL-6, CSF interleukin-6; NMDAR, N-methyl-D-aspartate receptor; MOG, myelin-oligodendrocyte glycoprotein; ADEM, acute disseminated encephalomyelitis; LE, limbic encephalitis; ANPRA, antibody-negative probable autoimmune encephalitis.
