# Supplementary material for: The role of CSF IL-6 levels in diagnosis and outcome prediction of autoimmune encephalitis
Source: Front Immunol. 2026 Jul 13;17:1865851. doi: 10.3389/fimmu.2026.1865851 (PMC13402481; doi:10.3389/fimmu.2026.1865851)
Supplement: Supplementary Table 1 — Distribution of CSF IL-6 levels across encephalitis subtypes. Associations between the subtypes of AE and CSF IL-6 are shown. CSF IL-6, CSF interleukin-6; NMDAR, N-methyl-D-aspartate receptor; MOG, myelin-oligodendrocyte glycoprotein; ADEM, acute disseminated encephalomyelitis; LE, limbic encephalitis; ANPRA, antibody-negative probable autoimmune encephalitis. [file Table1.docx]

Supplemental Table Distribution of CSF IL-6 levels across encephalitis subtypes

| **subtype** | | **median (pg/mL)** | **IQR** | **range** |
| --- | --- | --- | --- | --- |
| **Antibody-positive encephalitis** | **Anti-NMDAR encephalitis (n=14)** | 15.25 | 3.55–120.7 | 2.2–1060 |
|  | **Anti-MOG encephalitis (n=3)** | 16.5 | 11.3–75.3 | 6.0–134 |
|  | **Other antibody-positive encephalitis (n=8)** | 118.5 | 68.1–289.3 | 9.9–1880 |
| **Antibody-negative encephalitis** | **ADEM (n=5)** | 248 | 25.1–446.0 | 21.7–474 |
|  | **LE (n=3)** | 89.6 | 53.3–115.8 | 17.1–142 |
|  | **ANPRA (n=7)** | 39.3 | 11.4–166.9 | 3.7–931 |
|  | **Possible (n=15)** | 57.3 | 5.4–361.5 | 2.7–2220 |
